# Birt-Hogg-Dubé syndrome in two Chinese families with mutations in the *FLCN* gene

**DOI:** 10.1186/s12881-017-0519-z

**Published:** 2018-01-22

**Authors:** Xiaocan Hou, Yuan Zhou, Yun Peng, Rong Qiu, Kun Xia, Beisha Tang, Wei Zhuang, Hong Jiang

**Affiliations:** 1 Department of Neurology, Xiangya Hospital, Central South University, Changsha, Hunan People’s Republic of China; 2 School of Information Science and Engineering, Central South University, Changsha, Hunan People’s Republic of China; 3 Laboratory of Medical Genetics, Central South University, Changsha, Hunan People’s Republic of China; 4 Key Laboratory of Hunan Province in Neurodegenerative Disorders, Central South University, Changsha, People’s Republic of China; 5 Department of Thoracic Surgery, Xiangya Hospital, Central South University, Changsha, Hunan People’s Republic of China; 6 Xiangya Hospital, Central South University, 87 Xiangya, Kaifu, Changsha, Hunan province 410008 China; 7 National Institute of Geriatrics Clinical Research Center, Xiangya Hospital, Central South University, Changsha, Hunan People’s Republic of China

**Keywords:** Birt-Hogg-Dubé syndrome, *FLCN*, Pneumothorax

## Abstract

**Background:**

Birt-Hogg-Dubé syndrome is an autosomal dominant hereditary condition caused by mutations in the folliculin-encoding gene *FLCN* (NM_144997). It is associated with skin lesions such as fibrofolliculoma, acrochordon and trichodiscoma; pulmonary lesions including spontaneous pneumothorax and pulmonary cysts and renal cancer.

**Methods:**

Genomic DNA was extracted from peripheral venous blood samples of the propositi and their family members. Genetic analysis was performed by whole exome sequencing and Sanger sequencing aiming at corresponding exons in *FLCN* gene to explore the genetic mutations of these two families.

**Results:**

In this study, we performed genetic analysis by whole exome sequencing and Sanger sequencing aiming at corresponding exons in *FLCN* gene to explore the genetic mutations in two Chinese families. Patients from family 1 mostly suffered from pneumothorax and pulmonary cysts, several of whom also mentioned skin lesions or kidney lesions. While in family 2, only thoracic lesions were found in the patients, without any other clinical manifestations. Two *FLCN* mutations have been identified: One is an insertion mutation (c.1579_1580insA/p.R527Xfs on exon 14) previously reported in three Asian families (one mainland family and two Taiwanese families); while the other is a firstly reviewed mutation in Asian population (c.649C > T / p.Gln217X on exon 7) that ever been detected in a French family.

**Conclusions:**

Overall, The detection of these two mutations expands the spectrum of *FLCN* mutations and will provide insight into genetic diagnosis and counseling of Birt-Hogg-Dubé syndrome.

## Background

Birt-Hogg-Dubé syndrome (BHDS) is an autosomal dominant hereditary condition associated with skin lesions such as fibrofolliculoma, acrochordon and trichodiscoma, pulmonary lesions including spontaneous pneumothorax and pulmonary cysts and renal cancer. In 1925, Burnier and Rejsek reported an elderly female with multiple small skincolored papules on the head and neck, which was probably the first case of BHD [1]. In 1960, Zackheim and Pinkus described five more cases with similar clinical manifestations and histopathologic features [2]. In 1977, Birt, Hogg, and Dubé found that a few members of a thyroid cancer family had fibrofolliculoma that occurred in an autosomal dominant hereditary pattern [3]. In 2001, the susceptible locus was localised to chromosome 17p11.2 [4, 5]. Subsequently, protein-truncating mutations were identified in the *FLCN* (BHD) gene comprising 14 exons and encoding a protein called folliculin with unknown function [6]. Folliculin is expressed in most tissues including the skin and its appendages, the lungs (type 1 pneumocytes) and the kidney (distal nephron). Although the accurate function of this protein has not yet been clarified, it seems to be involved in the adenosine-monophosphate-activated protein kinase and mTOR pathways [7, 8]. Some studies have proved that downstream molecules of insufficient *FLCN* such as S6 kinase and hypoxia-inducible factor 1-alpha (HIF-1a) increases in renal tumors derived from BHDS patients. In the lung, cyst-lining cells were suggested to be activated due to their immunostaining positivity for phospho-mTOR and phospho-S6 ribosomal protein [9–12]. As neoplastic hyperplasia hardly occurs in cyst-lining cells, the mTOR pathway may be less distinctively detected in pulmonary cysts [11].

More than 200 mutations in the *FLCN* gene have been identifed, most of which are frameshift, nonsense, missense, or splice site mutations. The most common mutation in patients with Birt-Hogg-Dubé syndrome is c.1285dupC located in exon 11 [13–22], followed by c.1533_1536delGATG [12, 15, 23–25] and c.1278dupC [26–29] depending on literatures listed worldwide up to date. Table 1 presents the mutations described in the *FLCN* gene up to now according to literatures summarized by searching “Birt-Hogg-Dubé syndrome” and “*FLCN*” on pubmed and Embase line.

**Table 1:** Germline mutations in Birt-Hogg-Dubé syndrome

Exon/Intron	Nucleotide changes	Amino acid changes
Exon 1	Exon1 deletion	Splice mutation
Exon 1	c.-487G > C	Splice mutation
Exon 1	c.-302G > A	Splice mutation
Exon 1	c.-299C > T	Splice mutation
Exon 1	chr17:17080497_17087267del; 17084378_17084502invins	Splice mutation
Exon 1	chr17:17078506_17084897del	Splice mutation
Exon 1	chr17:17080610_17086298del; insCCATGGGGG	Splice mutation
Exon 2–5	c.-227-853_c.397-295del	Splice mutation
Exon 3	c.-90A > G	Splice mutation
Exon 3	c. − 84G > A	Splice mutation
Exon 4	c.1A > G	p.Met1Val
Exon 4	c.3delG	p.Met1Xfs
Exon 4	c.3G > A	p.Met1?
Exon 4	c.50G > C	p.Arg17Pro
Exon 4	c.57_58delCT	p.Phe20Xfs
Exon 4	c.59delT	p.Phe20Xfs
Exon 4	c.119delG	p.Gly40Xfs
Exon 4	c.145G > T	p.Glu49^a^
Exon 4	c.147delA	p.Glu49Xfs
Exon 4	c.157C > T	p.Gln53^a^
Exon 4	c.158delA	p.Gln53Xfs
Exon 4	c.214delA	p.Ser72Xfs
Exon 4	c.235_238delTCGG	p.Ser79Xfs
Exon 4	c.240delC	p.Asp80Xfs
Exon 4	c.241delA	p.Met81Xfs
Exon 5	Deletion of Exon 5	Protein truncation
Exon 5	c.252delC	p.Gly84Xfs
Exon 5	c.296delA	p.Asp99Xfs
Exon 5	c.319_320delGTinsCAG	p.Val107 deletion/ insertion
Exon 5	c.319_320delGTinsCAC	p.Val107 deletion/ insertion
Exon 5	c.323G > T (778G > T)	p.Ser108Ile
Exon 5	c.328C > T	p.Gln110^a^
Exon 5	c.332_349del(18nucleotides)	p.His111_Gln116delXfs
Exon 5	c.340dupC	p.His114Xfs
Exon 5	c.347dupA	p.Leu117Xfs
Exon 5	c.376delG	p.Val126Xfs
Exon 5	c.394G > A	p.Glu132Lys
Exon 6	c.402delC	p.Pro135Xfs
Exon 6	c.404delC	p.Pro135Xfs
Exon 6	c.420delC	p.Ile141fs
Exon 6	c.427_429delTTC	p.Phe143del
Exon 6	c.443_459delACGGCTTTGTGTTCAGC	p.His148_153SerdelXfs
Exon 6	c.469_471delTTC	p.Phe157Xfs
Exon 6	c.499C > T	p.Gln167^a^
Exon 6	c.510C > G	p.Tyr170^a^
Exon 6	c.510C > A	p.Tyr170^a^
Exon 6	c.553 T > C	p.Ser185Pro
Exon 6	c.563delT	p.Phe188Xfs
Exon 6	c.[564_565dupCC;566_577delTGCTGGGGAAGG]	p.Leu189Xfs
Exon 6	c.573_574delinsT	p.Lys192Xfs
Exon 6	c.581delG	p.Gly195Xfs
Exon 6	c.583G > T	p.Gly195^a^
Exon 6	c.584delG	p.Gly195Xfs
Exon 6	c.601C > T	p.Gln201^a^
Exon 6	c.610_611delinsTA	p.Ala204^a^
Exon 7	c.632 633delAGinsC	p.Glu211Xfs
Exon 7	c.637delT	p.Phe213Xfs
Exon 7	c.649C > T	p.Gln217^a^
Exon 7	c.655dupG	p.Ala219Xfs
Exon 7	c.658C > T	p.Gln220^a^
Exon 7	c.668delA	p.Asn223Xfs
Exon 7	c.689dupT	p.Leu230Xfs
Exon 7	c.671_672delCA	p.Thr224Xfs
Exon 7	c.715C > T	p.Arg239Cys
Exon 7	c.726A > T	NS
Exon 7	c.769_771delTCC	p.Ser257Xfs
Exon 7	c.770_772delCCT	p.Ser257Xfs
Exon 7	c.747_756insGTGATGACAA	p.Asn249Xfs
Exon 7	c.779G > A	p.Trp260^a^
Exons 7–14	c.675-?_c.^a^ +?del	
Exon 8	∆E8	p.Trp260Xfs
Exon 8	c.836_839delCCGA	p.Thr279Xfs
Exon 8	c.853C > T	p.Gln285^a^
Exon 9	c.887C > A	p.Ser296^a^
Exon 9	c.889_890delGA	p.Glu297Xfs
Exon 9	c.890_893del	p.Glu297Xfs
Exon 9	c.923_950dup	Frameshift
Exon 9	c.932_933delCT	p.Pro311Xfs
Exon 9	c.933delT	p.Val312Xfs
Exon 9	c.943 G > T	p.Glu315^a^
Exon 9	c.946_947delAG	p.Ser316Xfs
Exon 9	c.991_992dupTC	p.Leu332Xfs
Exon 9	c.997_998delTC	p.Ser333Xfs
Exon 9	c.997_998dupTC	p.Gly334Xfs
Exon 9	c.1013delG	p.Trp338Xfs
Exon 9	c.1015C > T	p.Gln339^a^
Exon 9	c.1018delC	p.Arg341Xfs
Exon 9	c.1021delC	p.Arg341Xfs
Exons 9–14	c.872-?_c.1740 +? del	Protein truncation
Exon 10	c.1063 1065delGTC	p.Val355Xfs
Exon 10	c.1067 T > C	p.Leu356Pro
Exon 10	c.1076delC	p.Pro359Xfs
Exon 10	c.1095C > G	NS
Exon 10	c.1117C > T	p.Gln373^a^
Exon 10	c.1127G > A	p.Trp376^a^
Exon 10	c.1153 C > T	p.Gln385^a^
Exon 10	c.1156_1175del	Frameshift
Exon 10	c.1156_1176del	Frameshift
Exon 10	c.1165G > T	p.Glu389^a^
Exon 10–11	c.1063-154_1300 + 410dup	Exon 10 deletion
Exon 11	c.1183_1198del	Frameshift
Exon 11	c.1198G > A	p.Val400Ile
Exon 11	c.1215C > G	p.Tyr405^a^
Exon 11	c.1219delA	p.Ser407Xfs
Exon 11	c.1228G > T	p.Glu410^a^
Exon 11	c.1252delC	p.Leu418Xfs
Exon 11	c.1269C > T	NS
Exon 11	c.1278dupC	p.His429Xfs
Exon 11	c.1278delC	p.His429Xfs
Exon 11	c.1285dupC	p.His429Xfs
Exon 11	c.1285delC	p.His429Xfs
Exon 11	c.1285C > T	p.His429Tyr
Exon 11	c.1286dupA	p.His429Xfs
Exon 11	c.1294_1298delTCCTC	p.Ser432Xfs
Exon 11	c.1300G > A	Splice mutation
Exon 11	c.1300G > C	Splice mutation
Exon 12	c.1301-7_1304del;1323delCinsGA	Frameshift
Exon 12	c.1303delT	p.Phe435Xfs
Exon 12	c.1305delT	p.Phe435Xfs
Exon 12	c.1318 1334dup	Frameshift
Exon 12	c.1323delCinsGA	p.His442Xfs
Exon 12	c.1333G > A	p.Ala445Thr
Exon 12	c.1335_1351dup	Frameshift
Exon 12	c.1337 1343dup	Frameshift
Exon 12	c.1340 1346dup	Frameshift
Exon 12	c.1347_1353dupCCACCCT	Frameshift
Exon 12	c.1372dup (1827insC)	p.Gln458Xfs
Exon 12	c.1379_1380delTC	p.Leu460Xfs
Exon 12	c.1389C > G	p.Tyr463^a^
Exon 12	c.1408_1418 insGGGAGCCCTGT	Frameshift
Exon 12	c.1426dupG	Frameshift
Exon 12	c.1429C > T	p.Arg477^a^
Exon 12	CCACCCT insertion	
Exon 13	c.1487_1490dup	Frameshift
Exon 13	c.1481A > G	p.Asn494Ser
Exon 13	c.1489_1490delGT	p.Val497Xfs
Exon 13	c.1490insCTGT	Frameshift
Exon 13	c.1522_1524del AAG	p.Lys508Xfs
Exon 13	c.1523A > G	p.Lys508Arg
Exon 13	c.1528_1530delGAG	p.Glu510Xfs
Exon 13	c.1533G > A	p.Trp511^a^
Exon 13	c.1533_1536delGATG	p.Trp511^a^Xfs
Exon 14	c.1539-?_c.1740 +? del	Exon14 deletion
Exon 14	c.1552delC	p.Leu518Xfs
Exon 14	c.1557delT	p.Phe519Xfs
Exon 14	c.1579_1580insA	p.Arg527Xfs
Exon 14	c.1579C > T	p.Arg527^a^
Exon 14	c.1597_1598delCA	p.Gln533Xfs
Exon 14	c.1645C > G	p.Leu549Val
Exon 14	c.1658G > A	p.Trp553^a^
Exon 14	c.1677G > A	NS
Exon 14	c.1715 + 16insC(14–22)	Splice mutation
Exon 14	c.1715 + 582 T > C	Splice mutation
Intron1	c.-228 + 1368G > T	Splice mutation
Intron1	c.-229 + 994A > G	Splice mutation
Intron3	c.-25 + 100C > G	Splice mutation
Intron3	c.1-64A > G	Splice mutation
Intron 4	c.249 + 1G > T	Splice mutation
Intron 4	c.250-2A > G	Splice mutation
Intron 4	c.250-1G > A	Splice mutation
Intron 5	c.396 + 1G > A	Splice mutation
Intron 5	c.396 + 59 T > C	Splice mutation
Intron 5	c.397-14C > T	Splice mutation
Intron 5	c.397-13G > A	Splice mutation
Intron 5	c.397-13_397-4delGGCCCTCCAG	Splice mutation
Intron 5	c.397-10_397-2delGTCCCTCCA	Splice mutation
Intron 5	c.397-7_399delcctccagGTC	Splice mutation
Intron 5	c.397-2A > C	Splice mutation
Intron 5	c.397-1G > C	Splice mutation
Intron 5	c.397-7_399del	Splice mutation
Intron5-Exon6	cctccagGTCdeletion	Splice mutation
Intron6	c.618 + 2 T > A	Splice mutation
Intron6	c.619-66C > T	Splice mutation
Intron6	c.619-1G > A	Splice mutation
Intron 7	c.779 + 1G > T	Splice mutation
Intron 7	c.779 + 113C > T	Splice mutation
Intron 7	c.780-1G > T	Splice mutation
Intron8	c.871 + 3_871 + 4delGAinsTCCAGAT	Splice mutation
Intron8	c.871 + 13 T > C	Splice mutation
Intron8	c.871 + 16 T > A	Splice mutation
Intron8	c.871 + 36G > A	Splice mutation
Intron8	c.871 + 204A > G	Splice mutation
Intron8	c.871 + 226G > A	Splice mutation
Intron8	c.871 + 684G > A	Splice mutation
Intron 9	c.1062 + 1G > A	Splice mutation
Intron 9	c.1062 + 2 T > G	Splice mutation
Intron 9	c.1062 + 5G > A	Splice mutation
Intron 9	c.1062 + 6C > T	Splice mutation
Intron 9	c.1062 + 47G > A	Splice mutation
Intron 9	c.1063-172C > G	Splice mutation
Intron 9	c.1063-117C > T	Splice mutation
Intron9	c.1063-10_1065delTCTTGTTTAGGTC	Exon 10 skip
Intron 9	c.1063-2A > G	Splice mutation
Intron 10	c.1176 + 31G > A	Splice mutation
Intron 10	c.1176 + 39G > A	Splice mutation
Intron 10	c.1176 + 68G > C	Splice mutation
Intron 10	c.1176 + 134G > C	Splice mutation
Intron 10	c.1176 + 179A > G	Splice mutation
Intron 10	c.1177-165C > T	Splice mutation
Intron 10	c.1177-8_1177-6delTCC	Splice mutation
Intron 10	c.1177-5_1177-3delCTC	Splice mutation
Intron10	c.1177-2A > G	Splice mutation
Intron 11	c.1300 + 2 T > C	Splice mutation
Intron 11	c.1301-59C > T	Splice mutation
Intron 11	c.1301-7del11; 1323delCinsGA	Splice mutation
Intron 12	c.1432 + 1G > A	Splice mutation
Intron 12	c.1432 + 4 C > T	Splice mutation
Intron 12	c.1433-38A > G	Splice mutation
Intron 12	c.1433-1G > T	Splice mutation
Intron 13	c.1538 + 121C > T	Splice mutation

NS represented that the mutation was synonymous and the amino acid was not changed

fs represented frameshift

^a^designates a stop codon

### Objectives

The aim of this study is to explore the genetic mutations of two suspected BHDS families, and to see if they could expand the spectrum of *FLCN* mutations.

## Methods

The two BHDS families were recruited from Peking Union Medical College Hospital and Xiangya Hospital Central South University. Detailed physical examination and other relevant examination of the participants were carried out. Peripheral venous blood samples of the participants were collected with anticoagulant tubes, storage and transportation of which were under the condition of 4 °C, then genomic DNA was extracted from blood samples within 6 h for further gene analysis: The whole blood and erythrocyte lysate were mixed thoroughly, kept still on ice for about 30 min until clear and then centrifuged at 3000 rpm for 10 min (4 °C); abandoned the supernatant, and mixed the remnant with nuclear lysate. Then added proteinase K into the mixture and mixed them thoroughly until there was no cell precipitate. Added SDS and shook at 37°Cfor 6 h or overnight. Added saturated phenol, mixed well up and down and centrifuged at 3000 rpm for 10 min (4 °C). Then put the supernatant into the mixture of saturated phenol and chloroform (1: 1), mixed well up and down and centrifuged at 3000 rpm for 10 min (4 °C); after that, put the supernatant into chloroform, mixed thoroughly up and down and centrifuged at 3000 rpm for 10 min (4 °C). The supernatant was added to a centrifuge tube previously charged with ethanol, gently inverted it to precipitate the DNA. The DNA and a small amount of ethanol was transferred to an eppendorf tube finally and stored at −20 °C in reserve.

With clinical manifestations and family history of pneumothorax, the patients and some of their relatives were diagnosed with suspected BHDS, at the meantime, unaffected relatives were invited to participate as controls. Members II10, III8, III10, III11, III12, III13, III14, IV1, IV2, IV3, IV4 in family 1 and II1, III2 in family 2 were sequenced. Publication of all the medical data has obtained consent of the participants, and the propositi consented on behalf of the deceased patients to both participate and to have their data published.

We selected one patient from each family respectively (IV3 in family 1 and III2 in family 2), carrying out whole exome sequencing for mutation detection: The 300 ng genomic DNA concentrations were sheared with Covaris LE220 Sonicator (Covaris) to target of 150-200 bp average size. DNA libraries were prepared using SureselectXT reagent kit (Agilent). The fragments were repaired the 3′ and 5′ overhangs using End repair mix (component of SureselectXT) and purified using Agencourt AMPure XP Beads (Beckman). The purified fragments were added with’A’ tail using A tailing Mix (component of SureSelectXT) and then ligated with adapter using the DNA ligase (component of SureselectXT). The adapter-ligated DNA fragments were amplified with Herculase II Fusion DNA Polymerase (Agilent). Finally, the pre-capture libraries containing exome sequences were captured using SureSelect capture library kit (Agilent). DNA concentration of the enriched sequencing libraries was measured with the Qubit 2.0 fluorometer dsDNA HS Assay (Thermo Fisher Scientific). Size distribution of the resulting sequencing libraries was analyzed using Agilent BioAnalyzer 2100 (Agilent). The libraries were used in cluster formation on an Illumina cBOT cluster generation system with HiSeq PE Cluster Kits (illumina). Paired-end sequencing is performed using an Illumina HiSeq system following Illumina-provided protocols for 2 × 150 paired-end sequencing. Then we applied Sanger sequencing aiming at corresponding exons in *FLCN* gene for subsequent validation of other family members roughly as follows: PCR amplification with appropriate primers on PCR amplifier - PCR cleanup in magnetic bead purification system - cycle sequencing on PCR amplifier - sequencing cleanup on magnetic bead purification platform - capillary electrophoresis on ABI3730. Interpretation of Sanger sequencing results was performed using SnapGene Software.

## Results

### Family 1 (F1)

**Fig. 1:**
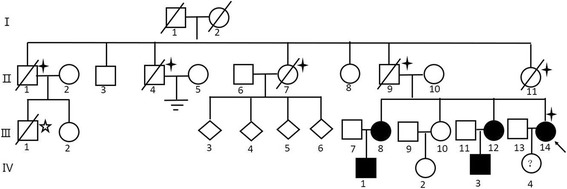
Pedigre of family 1.  proband.  Cases with stroke.  Thrombocythemia Case

**Fig. 2:**
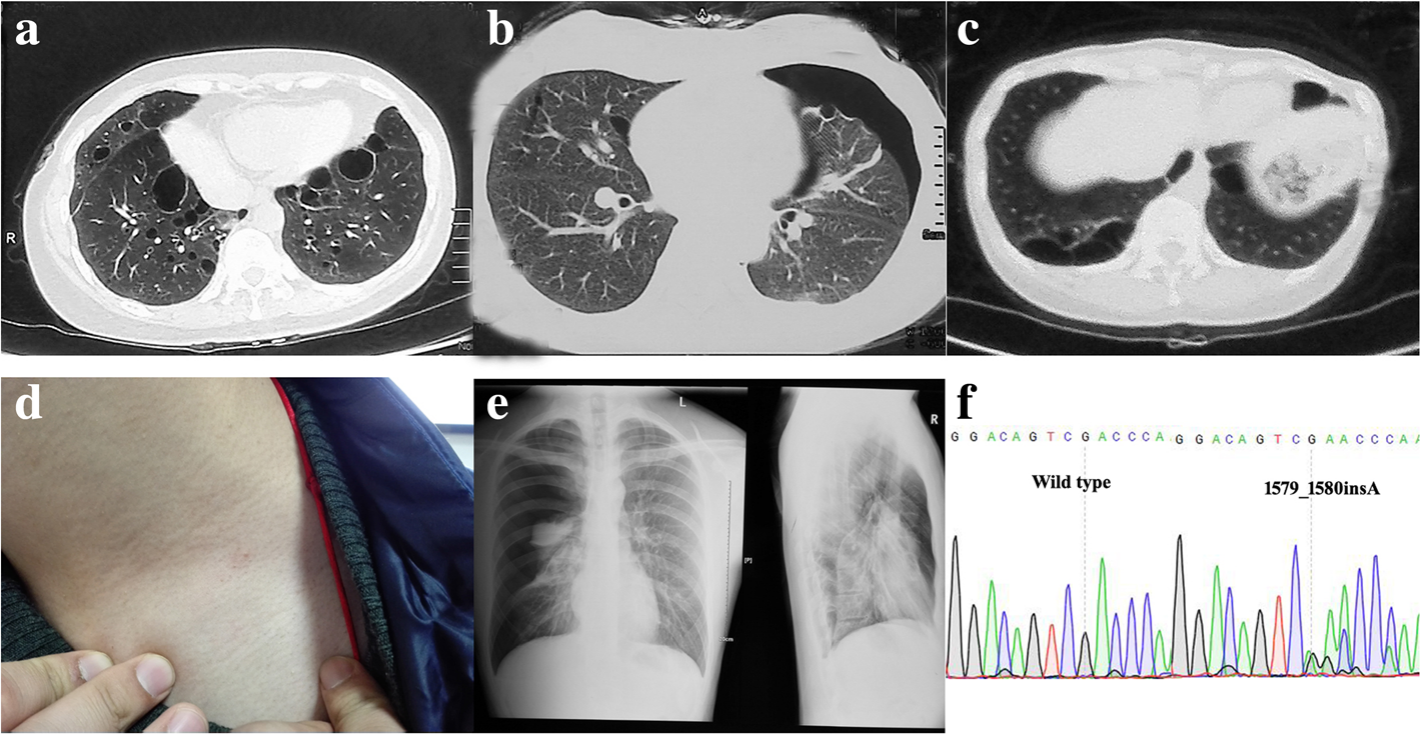
Examination results and Sequence diagram of family 1. **a**, **b**, **c** Computed tomography scans showing multiple cystic lesions in the lungs of patients (III8、III12、III14). **b**, **e** Computed tomography scan and X-ray examination results showing pneumothorax (III8、IV3). **d** Fat granules on the skin (IV1). **f** Direct sequencing of exon 14 of *FLCN* revealed the frameshift mutation: c.1579_1580insA on exon 14

The proband, a 47-year-old woman with a 25-year history of left-lung-pneumothorax, has had her left lung partially resected. Moreover, she was diagnosed with cerebral infarction 3 years ago on account of right limb numbness and visual defect in the lower half of the right eye. In addition, two of her sisters and their sons (Fig. 1: III8, III12, IV1, IV3) also had spontaneous pneumothorax history at the age of 39, 48, 21 and 21 respectively, a maximum frequency of which was six times. Diffuse lesions of the thyroid gland, superficial lymph node enlargement of the neck and extremities and subcutaneous nodules of the head, neck and hands were also revealed in one of her sister (III8) after pulmonary bubble resection; computed tomography (CT) scans of the other sister (III12) who had a history of hysteromyoma excision ever showed double renal cysts, which disappeared 2 years later in the renal ultrasonic examination results. While one nephew (IV1) of the proband had fat granules on his face and neck, who once underwent right branchial cystectomy; the other nephew (IV3) was diagnosed with chronic pancreatitis at 11 years old. A few of her other family members (Fig. 1:II1, II4, II7, II9, II11; II9: cerebral hemorrhage, others: cerebral infarction) also suffered from stroke, all of whom have passed away. One died of thrombocythemia (Fig. 1:III1). (Fig. 2).

### Family 2 (F2)

**Fig. 3:**
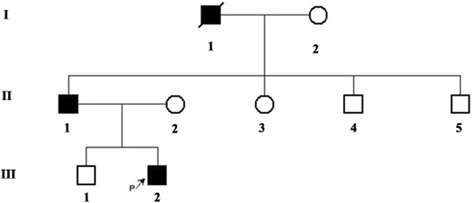
Pedigre of family 2.  proband

**Fig. 4:**
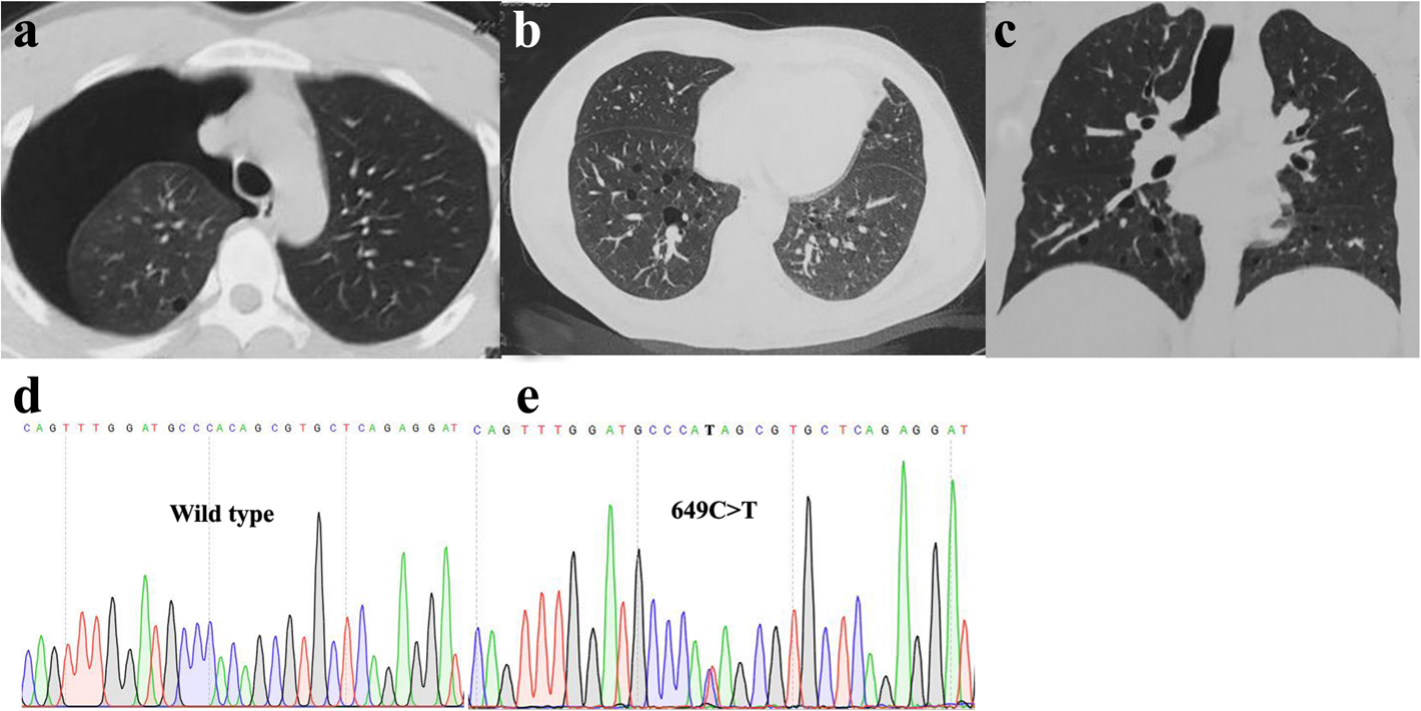
Examination results and Sequence diagram of family 2. **a** Computed tomography scans showing pulmonary cyst and pneumothorax (III2). **b**, **c** Multiple pulmonary cysts and pneumothorax in the lung of the proband’s father (II1). **d**, **e** Direct sequencing of exon7 of *FLCN* revealed the nonsense mutation: c.649C > T on exon 7

A 26-year-old man with after-exercise pectoralgia was diagnosed pneumothorax with CT scans, and before that, he once had a pneumothorax attack. In his family members, his father and grandfather also had pneumothorax history, for which his father had a thoracoscopic surgery. Besides, his grandfather passed away because of nephropathy without concrete information (Figs. 3 and 4). The clinical information of the two families are listed in Table 2.

**Table 2:** Summary of clinical information of the two families

Number	Family	Sex	Age	Pneumothorax	Pulmonary Cysts	Skin lesion	Kidney lesion	Mutation Region
III8	F1	Female	53	Yes	Yes	Subcutaneous nodule	No	Exon 14
III12	F1	Female	48	Yes	Yes	No	Renal cysts	Exon 14
III14	F1	Female	47	Yes	Yes	No	No	Exon 14
IV1	F1	Male	28	Yes	No	Fat granules	No	Exon 14
IV3	F1	Male	21	Yes	Yes	No	No	Exon 14
IV4	F1	Female	18	No	No	No	No	Exon 14
II1	F2	Male	52	Yes	Yes	No	No	Exon 7
III2	F2	Male	26	Yes	Yes	No	No	Exon 7

Mutation examinations revealed that the proband, her two sisters, two nephews (III8, III12, IV1, IV3) and her son (IV4) in F1 all carried a one-base (A) -insertion between nucleotides c.1579_1580 on exon 14 (c.1579_1580insA) (Fig. 2f), resulting in a frameshift mutation (p.Arg527Xfs), which has ever been reported in three Asian families [30–32]; while the proband and his father in F2 carried a one-base-substitution of C by T at nucleotide c.649 on exon 7 (c.649C > T) (Fig. 4d, e), resulting in a nonsense mutation (p.Gln217X), which was once recovered in a French family [22]. In addition, there are no mutations detected in the control subjects (II10, III10, III11, III13, IV2).

## Discussion

Studies of patients with Birt-Hogg-Dubé syndrome are very rare especially in Asian countries.

In this study, we described two BHDS families and applied whole exome sequencing and Sanger sequencing to explore the genetic mutations. Patients from family 1 mostly suffered from pneumothorax and pulmonary cysts, several of whom also mentioned skin lesions or kidney lesions. While in family 2, only thoracic lesions were found in the patients, without any other clinical manifestations. Two *FLCN* mutations have been identified: One is an insertion mutation (c.1579_1580insA/p.R527Xfs) previously reported in three Asian families (one mainland family and two Taiwanese families); while the other is a firstly reviewed mutation in Asian population (c.649C > T/p.Gln217X) that ever been detected in a french family.

As we have reported above, patients from these two families were mostly characterized by pneumothorax, and even without any other clinical manifestations, which may remind us of BHDS and carrying out genetic tests for patients with familial pneumothorax history. However, the exact mechanism of this syndrome is still unclear till now. Our study could only expand the spectrum of *FLCN* mutations ethnically, there are still many aspects of BHDS to be explored.

## Conclusions

Our detection of these two mutations expands the spectrum of *FLCN* mutations and will provide insight into genetic diagnosis and counseling of Birt-Hogg-Dubé syndrome.

## Abbreviation

BHDS: Birt-Hogg-Dubé syndrome
